# *Molecular docking* analysis of phytochemicals from *Soothaga Thadai Kudineer* with cyp-17α-hydroxylase enzyme

**DOI:** 10.6026/973206300210274

**Published:** 2025-02-28

**Authors:** Saraswathi Balasubramaniyan, Siva Annamalai, Deepa Ravichandran, Vinu Bharathi Balasubramaniam, Vinodini Ramamoorthy, Shunmugaram Shenbagaraj

**Affiliations:** 1 Department of Siddha Physician, Om Muruga Siddha Clinic, Thanjavur, Tamil Nadu-613006, India; 2 Department of Research Associate (S)-II, Siddha Regional Research Institute, Puducherry-13, India; 3 Department of Research Officer (S), Siddha Regional Research Institute, Puducherry-13, India

**Keywords:** *Soothaga Thadai Kudineer*, siddha medicine, *Molecular docking*

## Abstract

Polycystic ovarian syndrome (PCOS) affects 9.2% of women of reproductive age and has doubled in prevalence, rising from 6 million cases in 1990 to 12.13 million in 2019. PCOS is characterized by symptoms like irregular periods, acne, hirsutism, male-pattern baldness, weight gain, mood swings and infertility. PCOS is primarily associated with elevated androgen levels. The use of Indian *Soothaga Thadai Kudineer* for managing PCOS is well documented. Therefore, it is of interest to report the *Molecular docking* analysis of phytochemicals from Indian *Soothaga Thadai Kudineer* with cyp-17α-hydroxylase enzyme (CYP17). Analysis shows that phytochemicals such as gingerenone A, chlorogenic acid and piperine present in *Soothaga Thadai Kudineer* exhibit strong binding affinities to the enzyme suggesting their potential as CYP17 inhibitors for further validation and consideration.

## Background:

Polycystic ovary syndrome (PCOS) is a prevalent condition, affecting approximately 9.2% of women of reproductive age and is one of the leading causes of infertility. A study highlights the significant rise in the number of women diagnosed with PCOS, from 6 million in 1990 to 12.13 million in 2019, underscoring its growing impact on women's health [1, 2]. Hormonal imbalances, irregular periods, excess androgen levels and cysts in the ovaries are the features of PCOS [3]. Clinical symptoms include 'heavy, long, intermittent, unpredictable or absent periods, infertility, acne or oily skin, excessive hair on the face (Hirsutism) or body, male-pattern baldness or hair thinning, weight gain especially around the belly, mood swings' [4] and leads to other health conditions including 'Type 2 Diabetes Mellitus, Hypertension, Dyslipidaemia and heart disease' [5]. The primary symptom of PCOS in the majority of patients is elevated androgen levels. A particular androgen-regulating protein, P450 17α hydroxylase, is encoded by the promoter region of the enzyme CYP 17. This protein is crucial for both obesity and reproductive function [6]. The microsomal enzyme CYP17 is required for the synthesis of gonadal and adrenal steroids. It possesses lyase and hydroxylase functions, also expresses itself in gonadal tissues as well as the adrenal cortex's zona fasciculata and zona recticulars. Four major steroid hormones, including cortisol, testosterone, estradiol and DHEA, are formed primarily by CYP17 alpha. Firstly it acts on progesterone and pregnenolone at the C17 position, resulting in hydroxylation to form '17-hydroxypregnenolone' and '17-hydroxyprogesterone'. It then splits the 'C17-C20 bond of 17-hydroxypregnenolone' and '17-hydroxyprogesteron' to form androstenedione and dehydroepiandrosterone [7]. An imbalance of steroidogenesis in the adrenal glands and ovaries causes hypoandrogenism in PCOS. Androgen is converted to testosterone more quickly as a result of the overexpression of the CYP17 encoding gene [8]. The use of the Indian *Soothaga Thadai Kudineer* for managing PCOS is well documented. Therefore, it is of interest to report the *Molecular docking* analysis of phytochemicals from Indian *Soothaga Thadai Kudineer* with cyp-17α-hydroxylase enzyme (CYP17).

## Materials and Methods:

## Study drug:

The Ingredients and Phytochemicals of Siddha Polyherbal formulation Soothaka Thadai Kudineer [9, 10, 11, 12, 13, 14, 15, 16, 17-18] were tabulated in Table 1.

## Target protein preparation:

The protein data bank was used to obtain the crystalline structure of the target protein enzyme, CYP-17α-hydroxylase (Figure 1 - see PDF), with PDB number 3RUK. A protein clean-up procedure was then carried out and the necessary missing hydrogen atom was added.

## Ligand preparation:

The Auto-dock program was used to assess how differently oriented the lead molecules were in relation to the target proteins and the interaction study analysis was used to determine which dock posture was optimal. 2D and 3D structures of ligands were depicted in Figure 2 (see PDF) and their properties were tabulated in Table 2.

## Docking procedure:

For the extracted phytocomponents, docking computations were performed against the target enzyme, CYP-17α-hydroxylase. Using Auto Dock Tools, essential hydrogen atoms, Kollman united atom type charges and solvation parameters were incorporated. Affinity (grid) maps of xx Å grid points and 0.375 Å spacing were generated using the Auto grid program [19]. The dielectric functions dependent on distance and the Auto Dock parameter set were utilized to compute the electrostatic and van der Waals terms, respectively. Using the Solis & Wets local search technique and the Lamarckian genetic algorithm (LGA), docking simulations were carried out [20]. The ligand molecules' initial orientation, location as well as and torsions were all randomly determined. During docking, all rotatable torsions were freed. Every docking experiment was the result of two separate runs that were intended to end after a maximum of 250000 energy assessments. 150 were the target population. A translational step of 0.2 Å and quaternion and torsion steps of 5 were used in the search.

## Results:

The *Molecular docking* analysis of nine compounds against CYP-17α-hydroxylase (PDB ID: 3RUK) reveals varying levels of binding affinities and interactions with specific amino acid residues. Gingerenone-A showed the highest binding affinity, with an estimated free energy of -9.07 kcal/mol and an inhibition constant (Ki) of 224.04 nM, suggesting it could be a potent inhibitor. Compounds like Chlorogenic acid and Piperine also demonstrated strong binding with inhibition constants in the nanomolar range, indicating significant inhibitory potential. Kaempferitrin and Oleic acid, on the other hand, displayed much weaker affinities, with higher Ki values, suggesting limited effectiveness as inhibitors. Across these compounds, residues such as ALA, THR, VAL and ILE were frequently involved, suggesting their importance in the ligand binding and stabilization processes within the enzyme's active site. The compounds also varied in their electrostatic and total intermolecular energies, with interaction surfaces ranging from 303.81 to 734.107, reflecting differences in the extent of enzyme surface engagement. This study highlights Gingerenone-A as a promising candidate for further exploration as a CYP-17α-hydroxylase inhibitor, with Chlorogenic acid and Piperine also showing potential as effective inhibitors. The docking poses, 2D Interaction Plot Analysis and Hydrogen bond plotting were depicted in Figure 3 (see PDF), Figure 4 (see PDF), & Figure 5 (see PDF) respectively. The results of the *Molecular docking* were tabulated in Table 3 & Table 4.

## Discussion:

Polycystic ovarian syndrome is treated with a variety of synthetic hormones like Oral Contraceptive Pills (OCP) which leads to adverse effects like venous thromboembolism (VTE), Thrombolytic shock and Myocardial Infarction [21]. Therefore, there is a need for alternative intervention to treat the ailments. The findings of this study demonstrate the potential of *Soothaga Thadai Kudineer*, a Siddha polyherbal formulation, in modulating the activity of the enzyme CYP17α-hydroxylase, which plays a key role in androgen biosynthesis.

Elevated levels of androgens are a primary characteristic of PCOS and CYP17α-hydroxylase is critical in regulating steroidogenesis in the ovaries and adrenal glands. The *Molecular docking* analysis presented in this study highlights several phytochemicals that exhibit significant binding affinities to CYP17α-hydroxylase, which suggests that they may serve as potential inhibitors of the enzyme's activity, thereby mitigating the excessive androgen production in PCOS.

Among the nine phytochemicals tested, Ginger none-A, Chlorogenic acid and Piperine exhibited the most promising interactions, with low estimated binding energies and inhibition constants (Ki). This indicates a strong binding affinity to the target protein, suggesting that these compounds may act as effective inhibitors of CYP17α-hydroxylase.

Ginger none-A demonstrated the highest binding energy (-9.07 kcal/mol) and the lowest inhibition constant (224.04 nM), making it a prime candidate for further investigation as a natural therapeutic agent for managing PCOS. The interaction of phytochemicals with key amino acid residues of the enzyme, particularly those located in the active site, further supports their potential inhibitory role. Notably, the amino acid residues ALA302, THR306, VAL366 and ILE371 were consistently involved in the binding of several phytochemicals, suggesting that these residues play a crucial role in the enzyme's catalytic function. By forming stable hydrogen bonds or hydrophobic interactions with these residues, the phytochemicals may hinder the enzyme's ability to catalyse steroid genesis, thus reducing androgen production. The presence of diverse bioactive compounds in *Soothaga Thadai Kudineer* indicates its potential as a multitarget therapeutic for PCOS. The polyherbal nature of this formulation could allow for a synergistic effect, where multiple phytochemicals work together to inhibit CYP17α-hydroxylase, also potentially addressing other factors of PCOS, such as inflammation, oxidative stress and insulin resistance [22].

For instance, Kaempferitrin and Chlorogenic acid have known antioxidant properties, which could further benefit individuals with PCOS by reducing oxidative damage and improving metabolic function [23, 24]. Also some studies reported that the drug *Soothaga Thadai Kudineer* has been found to be effective in treating Secondary Amenorrhoea and reduces the premenstrual symptoms like back pain, abdominal bloating, headache, mood swing, irritability and tension [25, 26].

However, it is important to note that while in silico docking studies provide valuable insights into the potential interactions between phytochemicals and target proteins, these results need to be validated through *in vitro* and *in vivo* studies. Further experimental research is necessary to confirm the inhibitory effects of these phytochemicals on CYP17α-hydroxylase activity in biological systems, as well as their efficacy in reducing androgen levels in PCOS patients. Additionally, the safety and bioavailability of these compounds need to be assessed to ensure that they can be effectively used as therapeutic agents.

## Conclusion:

The use of Indian *Soothaga Thadai Kudineer* as a natural treatment for PCOS by targeting CYP17α-hydroxylase is shown. Further, phytochemicals such as gingerenone-A, chlorogenic acid and piperine present in *Soothaga Thadai Kudineer* exhibit strong binding affinities to the enzyme suggesting their potential as CYP17 inhibitors. It should be noted that *in vitro* and *in vivo* analysis are required for validation and consideration.

## Ethical approval:

The authors declare that due to an *In-silico* analysis this study does not need any ethical approval.

## Consent to participate:

Not applicable

## Consent to publish:

Not applicable

## Author contributions:

All authors have equally contributed in the manuscript preparation like concept, designing and collection of the data, as well as the writing and revision of the article.

## Funding:

The authors declare that no funds, grants, or other support were received during the preparation of this manuscript.

## Availability of data and materials:

All the data's are available with authors upon reasonable request.

## Source of funding:

No funds, grants, or other support was received for this work.

## Figures and Tables

**Table 1:** Ingredients of the Soothaka Thadai Kudineer and their selected phytochemicals

**S. No**	**Ingredients**		**Family**	**Phytochemicals**
	**Botanical Name**	**Vernacular Name**		
1.	*Trachyspermum ammi* Linn.	*Omam*	Apiaceae	Carvone [10]
2.	*Bambusa arundinacea* Linn.	*Munkililai*	Poaceae	Chlorogenic acid [11]
3.	*Crataeva religiosa* G.Frost.	*Mavilinkappattai*	Capparaceae	β-caryophyllene [12]
4.	*Smilax china* Linn.	*Parankipattai*	Liliaceae	Kaempferitrin [13]
5.	*Zingiber officinale* Roscoe.	*Cukku*	Zingiberacea	Gingerenone-A [14]
6.	*Piper longum* Linn.	*Tippili*	Piperaceae	Piperine [15]
7.	*Plumbago indica* Linn.	*Cittiramulaver, verpattai*	Plumbaginaceae	Plumbagin [16]
8.	*Nigella sativa* Linn.	*Karuñcirakam*	Ranunculaceae	Oleic acid [17]
9.	*Anethum graveolens* Linn.	*Catakuppai*	Apiaceae	Anethole [18]

**Table 2:** Ligand properties of the compounds selected for docking analysis

**S. No**	**Compound**	**Molar weight g/mol**	**Molecular Formula**	**H Bond Donor**	**H Bond Acceptor**	**Rotatable bonds**
1.	Carvone	150.221 g/mol	C_10_H_14_O	0	1	1
2.	Chlorogenic acid	354.31 g/mol	C_16_H_18_O_9_	6	9	5
3.	β-caryophyllene	204.35 g/mol	C_15_H_24_	0	0	0
4.	Kaempferitrin	578.5 g/mol	C_27_H_30_O_14_	8	14	5
5.	Gingerenone-A	356.4 g/mol	C_21_H_24_O_5_	2	5	9
6.	Piperine	285.34 g/mol	C_17_H_19_NO_3_	0	3	3
7.	Plumbagin	188.182 g/mol	C_11_H_8_O_3_	1	3	0
8.	Oleic acid	282.5 g/mol	C_18_H_34_O_2_	1	2	15
9.	Anethole	148.20g/mol	C_10_H_12_O	0	1	2

**Table 3:** Molecular docking against cyp- 17α-hydroxylase

**S. No**	**Compound**	**Est. Free Energy of Binding**	**Est.Inhibition Constant, Ki**	**Electrostatic Energy**	**Total Intermolec. Energy**	**Interact. Surface**
1.	Carvone	-6.17 kcal/mol	29.93 uM	-0.03 kcal/mol	-6.47 kcal/mol	362.976
2.	Chlorogenic acid	-8.43 kcal/mol	663.72 nM	-0.08 kcal/mol	-7.86 kcal/mol	536.859
3.	β-caryophyllene	-8.27 kcal/mol	872.02 nM	-0.01 kcal/mol	-8.27 kcal/mol	430.14
4.	Kaempferitrin	-4.19 kcal/mol	845.07 uM	-0.26 kcal/mol	-4.22 kcal/mol	734.107
5.	Gingerenone-A	-9.07 kcal/mol	224.04 nM	-0.18 kcal/mol	-8.18 kcal/mol	555.376
6.	Piperine	-8.53 kcal/mol	559.93 nM	-0.04 kcal/mol	-8.10 kcal/mol	456.344
7.	Plumbagin	-6.14 kcal/mol	31.59 uM	-0.03 kcal/mol	-6.44 kcal/mol	369.403
8.	Oleic acid	-3.94 kcal/mol	1.29 mM	-0.01 kcal/mol	-4.24 kcal/mol	303.81
9.	Anethole	-5.25 kcal/mol	142.87 uM	-0.12 kcal/mol	-5.84 kcal/mol	378.878

**Table 4:** The phytochemicals interact with amino acid residues to inhibit cyp-17α-hydroxylase

**Compounds**	**Interaction**	**Amino Acid Residues**															
Carvone	0	302	306	366	367	371	483										
		ALA	THR	VAL	ALA	ILE	VAL										
Chlorogenic acid	2	105	113	114	205	209	239	298	302	305	306	366	367	482	483		
		ALA	ALA	PHE	ILE	LEU	ARG	ASP	ALA	GLU	THR	VAL	ALA	VAL	VAL		
β-caryophyllene	0	302	306	366	367	371	482	483									
		ALA	THR	VAL	ALA	ILE	VAL	VAL									
Kaempferitrin	3	105	113	114	201	202	205	209	239	294	298	302	305	306	366	371	483
		ALA	ALA	PHE	TYR	ASN	ILE	LEU	ARG	THR	ASP	ALA	GLU	THR	VAL	ILE	VAL
Gingerenone-A	2	113	114	202	205	206	209	239	298	302	305	306	482	483			
		ALA	PHE	ASN	ILE	ILE	LEU	ARG	ASP	ALA	GLU	THR	VAL	VAL			
Piperine	0	113	114	302	305	306	371	483									
		ALA	PHE	ALA	GLU	THR	ILE	VAL									
Plumbagin	0	113	114	302	306	366	367	371	482								
		ALA	PHE	ALA	THR	VAL	ALA	ILE	VAL								
Oleic acid	2	201	202	205	239	297	298										
		TYR	ASN	ILE	ARG	GLY	ASP										
Anethole	0	113	298	302	306	366	367	371									
		ALA	ASP	ALA	THR	VAL	ALA	ILE									
